# Gene Signature Associated with Ferroptosis for Predicting Overall Survival among Ovarian Cancer Patients

**DOI:** 10.7150/jca.116777

**Published:** 2025-10-20

**Authors:** Yanling Li, Yidi Wang, Han Lei, Kai Wai Li, Jingman Tang, Lu Xu, Yan Liu, Jianhong Lu, Yulong Peng, Lili Fan, Xiaojuan Li, Jianbo He

**Affiliations:** 1 Hunan Cancer Hospital and The Affiliated Cancer Hospital of Xiangya School of Medicine, Central South University, Changsha 410000, China.; 2 Guangzhou Key Laboratory of Formula-Pattern of Traditional Chinese Medicine, School of Traditional Chinese Medicine, Jinan University, Guangzhou 510632, China.; 3 Department of Pathology, Xiangya Hospital, Xiangya School of Basic Medical Sciences, Central South University, Changsha 410000, China.; 4 School of Traditional Chinese Medicine, Jinan University, Guangzhou 510632, China.; 5 Department of Pathology, First Affiliated Hospital of Jinan University, Guangzhou 510630, China.; 6 State Key Laboratory of Dampness Syndrome of Chinese Medicine, the Second Affiliated Hospital of Guangzhou University of Chinese Medicine, Guangdong Provincial Hospital of Chinese Medicine, Guangzhou 510120, China.

**Keywords:** ovarian cancer, ferroptosis, overall survival prediction, prognostic signature.

## Abstract

Distinct from apoptosis, ferroptosis is intricately associated with intracellular iron ions and oxidative stress, representing a unique form of cell demise. In the treatment of ovarian cancer (OC), it assumes a crucial role, as research suggests its association with patient prognosis. This investigation delves into the correlation between genes associated with ferroptosis and the prognosis of OC, providing insights into its pathogenesis. Through the examination of mRNA expression using TCGA, ICGC, and GTEx databases, we identified a set of five pivotal genes (CD44, FTH1, ALOX12, SLC7A11, CRYAB) forming a prognostic model. Their regulation affects various aspects of OC, including the cell cycle, proliferation, invasiveness, immune response, and drug tolerance. To summarize, ferroptosis significantly impacts the prognosis of OC, and the targeting of relevant pathways holds potential for enhancing treatment outcomes, thereby guiding future research and personalized therapeutic strategies.

## Introduction

A prevalent and deadly form of cancer, ovarian cancer (OC) impacts the female reproductive system, accounting for approximately 4% of cancer-related fatalities in women globally. By reason of the small size of the ovary and its deep location within the pelvic cavity, OC often lacks noticeable symptoms, making it difficult to detect in its early stages. More than 80% of OC cases are diagnosed when the cancer has already advanced. Additionally, recurrence happens in over 70% of patients after receiving treatment 1, 2. The main method for treating OC involves a mix of surgery and chemotherapy. Nonetheless, the effectiveness of chemotherapy frequently encounters obstacles attributed to the emergence of drug resistance and adverse reactions. Therefore, identifying new biomarkers and treatment targets for OC is pivotal.

Ferroptosis, a variant of programmed cell death, entails the iron-dependent breakdown of lipids, leading to the buildup of lipid reactive oxygen species (L-ROS) 3-6. Over the past few years, stimulating ferroptosis to trigger cancer cell demise has surfaced as a promising therapeutic strategy 7-9. Previous studies have indicated that prolonged iron exposure constitutes a notable risk factor for the initiation and advancement of OC, with ferroptosis serving as a critical element in this scenario 10. Certain genes, such as p53 11, hTERT 12, TFR1, and FPN 13 are involved in the process of ferroptosis. However, the extent of the association between genes linked to ferroptosis and the future outcome of individuals with ovarian cancer remains largely uncertain.

For the present investigation, initially, we obtained mRNA expression data and corresponding clinical information of OC patients from publicly available databases. Subsequently, we formulated a multi-gene prognostic signature utilizing differentially expressed genes (DEGs) associated with ferroptosis in the TCGA cohort and validated its reliability in the ICGC cohort 14. In conclusion, we carried out studies on the enrichment of functional annotations to investigate the fundamental mechanisms. Our results revealed a new gene signature linked to ferroptosis, providing predictive significance for overall survival (OS) among OC patients. Additionally, the findings imply that addressing ferroptosis could present a potential therapeutic approach for OC.

## Results

**Figure 1:**
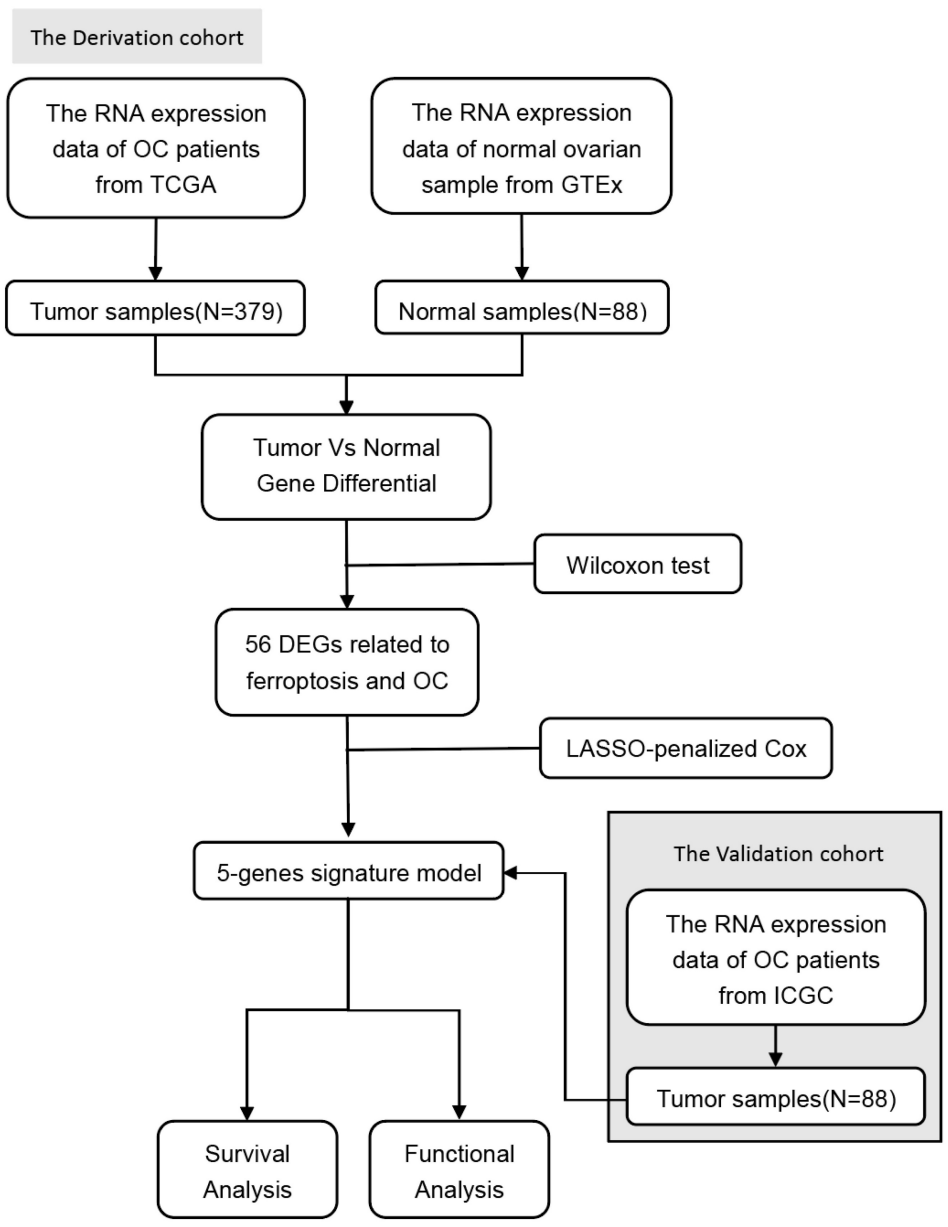
** Flowchart of data collection and analysis**.

The study's flow chart is depicted in Figure 1. In the analysis, we incorporated 379 patients with OC from the TCGA cohort, 108 patients with OC from the ICGC cohort, and 88 samples of normal ovarian tissue from the GTEx dataset.

### Identification of prognostic ferroptosis-related DEGs in the TCGA cohort

**Figure 2:**
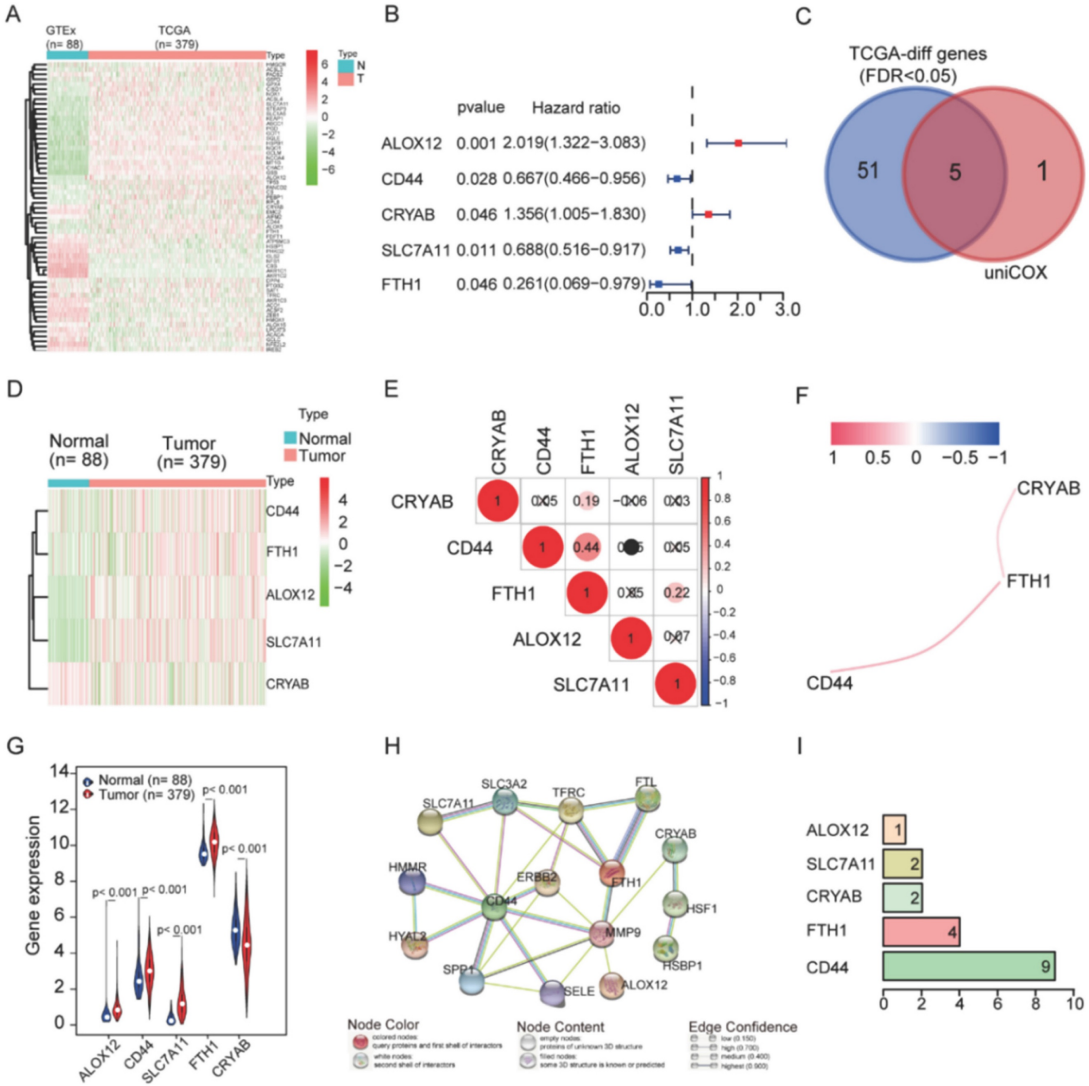
** Identification of the candidate ferroptosis-related genes in the TCGA cohort. A**. The heatmap of 56 ferroptosis-related genes were differentially expressed in the normal and tumor groups.** B**. Forest plots showing the results of the univariate Cox regression analysis between gene expression and OS. **C**. Venn diagram to identify differentially expressed genes between tumor and adjacent normal tissue that were correlated with OS. **D**. The heatmap of 5 ferroptosis-related genes were correlated with OS. **E**. The correction between hub-genes. **F**. The co-expression network between hub-genes. **G**. The violin plot of differently expressed genes in the normal and tumor groups. **H**. The network of hub-genes related genes with the function enrichment.** I**. The hub-genes related genes number.

Fifty-six ferroptosis-related genes showed significant differential expression between OC patients and normal controls (Figure 2A). In the univariate Cox regression analysis, five of them exhibited an association with OS. These five prognostics ferroptosis-related DEGs were kept (all FDR < 0.05, Figure 2B and 2C). Subsequently, we presented the distinct expression levels of the five genes within two distinct groups (Figure 2D and 2G). To understand the interactions among the five genes, we examined their correlations (Figure 2E and 2F) and interactions (Figure 2H and 2I). FTH1 expression was positively correlated with CD44, CRYAB, and SLC7A11 in OC, but not with ALOX12 (Figure 2E and 2F). Interestingly, CD44 was predicted to interact with SLC7A11 (Figure 2H), but there was no correlation between their expression levels in OC (Figure 2E). Protein-protein interaction network analysis showed that CD44 and FTH1 had more connections with other regulators, while ALOX12, SLC7A11, and CRYAB had fewer interactions with others (Figures 2H and 2I). FTH1 and CD44 were the hub genes in the interaction network.

### Classification of tumors was conducted based on the five ferroptosis-related genes

Based on their expression patterns, the five genes could be divided into two groups. One group (including CD44, FTH1, ALOX12, and SLC7A11) showed high expression in tumors, while the other gene (CRYAB) was abundant in normal tissues.

**Figure 3:**
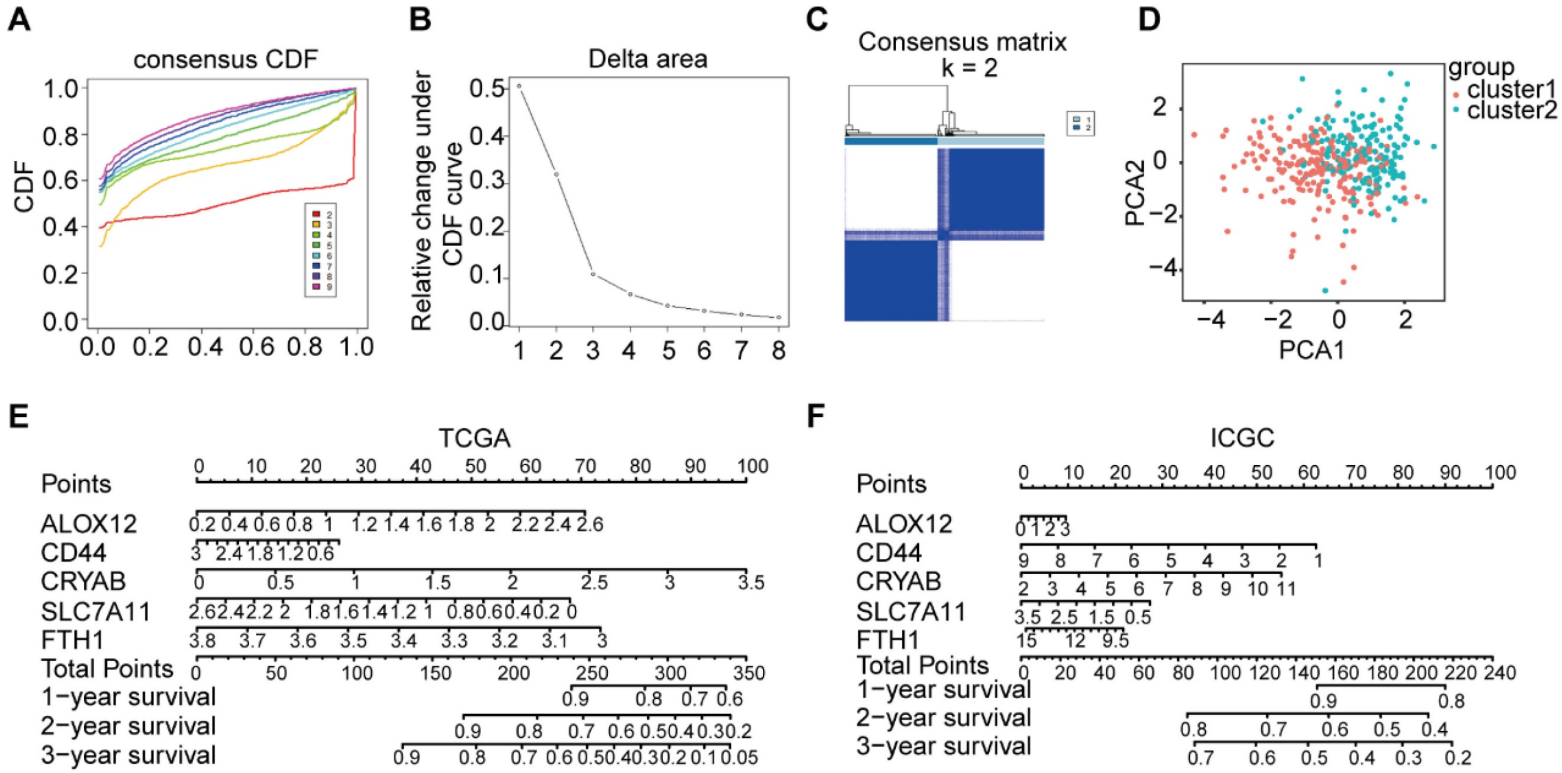
** The consensus clustering of the tumor classification and based on the 5-genes tumor were divided into high-risk and low-risk groups. A-B**. The corresponding relative change in area under the cumulative distribution function (CDF) curves when the cluster number changes from k to k+1. The range of k changed from 1 to 8, and the optimal k = 2. **C**. The consensus matrices are represented as heat map for the chosen optimal cluster number (k = 2) for the TCGA cohort. **D**. PCA plot of the TCGA cohort. **E-F**. The nomograph of TCGA and ICGC. The expression levels of 5 genes were detected, the scores were calculated, and then the total scores were calculated to predict the 1-3-year survival rate.

To explore the correlation between the expression patterns of ferroptosis-related genes and the prognosis of OC, we conducted consensus clustering on all 379 OC cases using the impartial expression data of all ferroptosis-related genes. As the stability of clustering increased from k = 1 to 8 in the TCGA database, k = 2 appeared as the optimal choice, considering the expression similarity of ferroptosis-related genes (Figure 3A-C). Subsequently, PCA was utilized to compare the transcriptional profiles between cluster 1 and cluster 2 groups, thereby confirming the rationale behind this grouping (Figure 3D). Moreover, a nomogram was constructed to forecast the survival probability of OC patients at 1-3 years, derived from the outcomes of the multivariate Cox regression analysis (Figure 3E-F).

### Development of a prognostic model was undertaken in both the TCGA and ICGC cohorts

In OC, we identified a total of 56 ferroptosis-related genes, of which five genes (CD44, FTH1, ALOX12, SLC7A11, and CRYAB) were significantly associated with the prognosis of OC. LASSO Cox regression analysis was employed to construct a prognostic model incorporating the five genes, aiming to enhance the prediction of clinical outcomes in OC characterized by abnormal expression of ferroptosis-related genes. Subsequently, a five-gene signature was formulated based on the optimal λ value. The risk score for the signature was computed using the following formula:



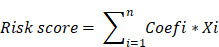



**Figure 4:**
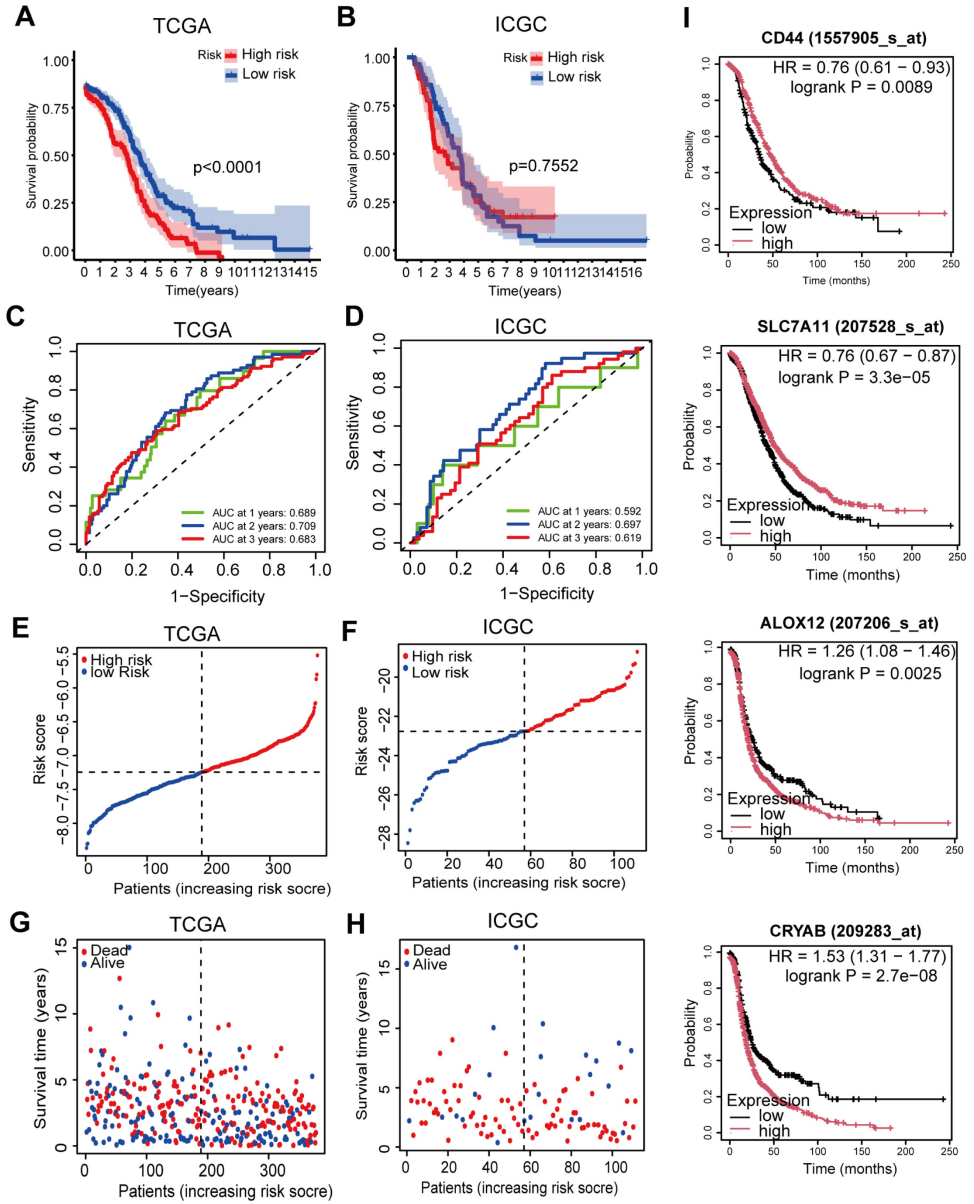
** Prognostic analysis of the 5-genes signature model in the TCGA and ICGC cohorts. A-B**. Kaplan-Meier curves for the OS of patients in high-risk and low-risk groups in TCGA and ICGC cohorts. **C-D**. AUC of time-dependent ROC curves verified the prognostic performance of the risk score in TCGA and ICGC cohorts. **E-F**. The distribution and median value of the risk scores in TCGA and ICGC cohorts. **G-H**. Survival status of patients in TCGA and ICGC cohorts. **I.** KM survival curves for OS in OC patients according to the tumor expression of key genes.

Where “Coefi” represents the coefficient, and “Xi” signifies the z-score transformed relative expression value of each selected gene. OC patients were stratified into a high-risk group (comprising samples with a risk score exceeding the median value) and a low-risk group (comprising samples with a risk score below the median value) based on their tumor sample's risk score. The comparison of OS between the two groups in the TCGA and ICGC cohorts revealed that patients in the high-risk group exhibited a significantly worse OS than their low-risk counterparts in the TCGA cohort (*P* < 0.0001, Figure 4A). However, no significant difference was observed in the ICGC cohort (*P* = 0.7552, Figure 4B). ROC analysis was conducted to evaluate the sensitivity and specificity of survival prediction according to the risk score, and the AUC values were derived from the ROC curves (Figure 4C and 4D). In the TCGA cohort, the AUC of the five-gene signature stood at 0.689 at one year, 0.709 at two years, and 0.683 at three years (Figure 4C). The AUC of the five-gene signature in the ICGC cohort was 0.592 at one year, 0.697 at two years, and 0.619 at three years (Figure 4D), signifying an acceptable predictive performance of the signature. Although the predictive performance declined somewhat in the ICGC cohort, the results maintain biological plausibility and show consistent trends with the TCGA cohort 15, 16.

**Figure 5:**
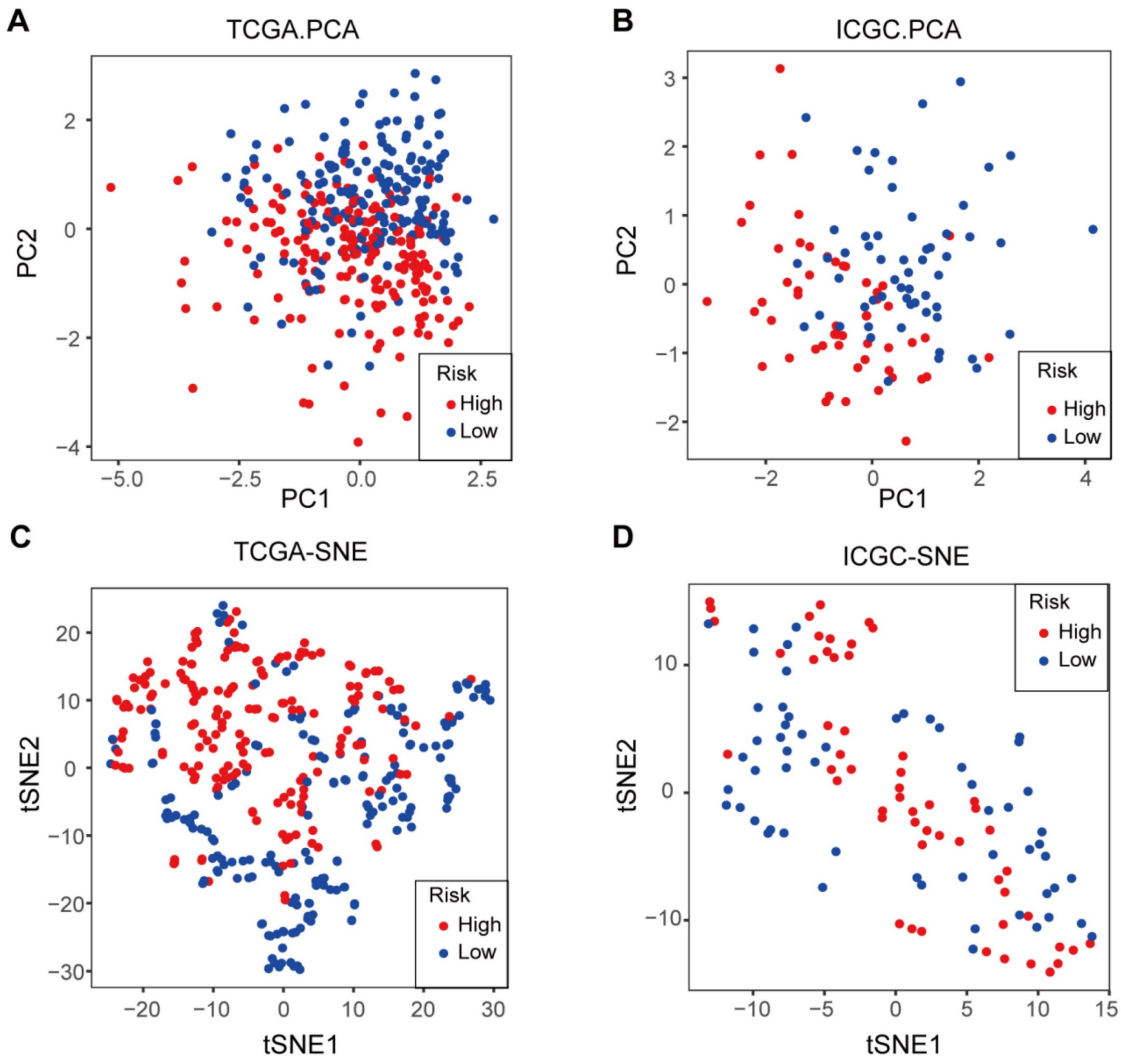
** The PCA plot and t-SNE analysis of TCGA and ICGC cohorts. A**. PCA plot of the TCGA cohort. **B**. PCA plot of the ICGC cohort. **C**. t-SNE analysis of the TCGA cohort. **D**. t-SNE analysis of the ICGC cohort.

Furthermore, an investigation was conducted into the distribution patterns of risk scores and survival statuses within the TCGA and ICGC cohorts (Figure 4E-F). Next, survival plots were produced for the TCGA and ICGC cohorts (Figure 4G-H). Then, to further validate the prognostic significance of the candidate genes, we performed survival analysis using the Kaplan-Meier Plotter database. The results demonstrated that high expression of CD44 (*P* = 0.0089) and SLC7A11 (*P* = 3.3×10^-5^) was significantly associated with better OS, whereas high expression of ALOX12 (*P* = 0.0025) and CRYAB (*P* = 2.7×10^-8^) correlated with poorer prognosis (Figure 4I). For further investigation, we conducted principal component analysis (PCA) and t-SNE analyses to compare the transcriptional profiles between cohorts categorized into elevated-risk and diminished-risk clusters in both the TCGA and ICGC datasets. The results demonstrated notable separation in opposite directions (Figure 5A-D).

### Analyses of functionality within the TCGA and ICGC cohorts

**Figure 6:**
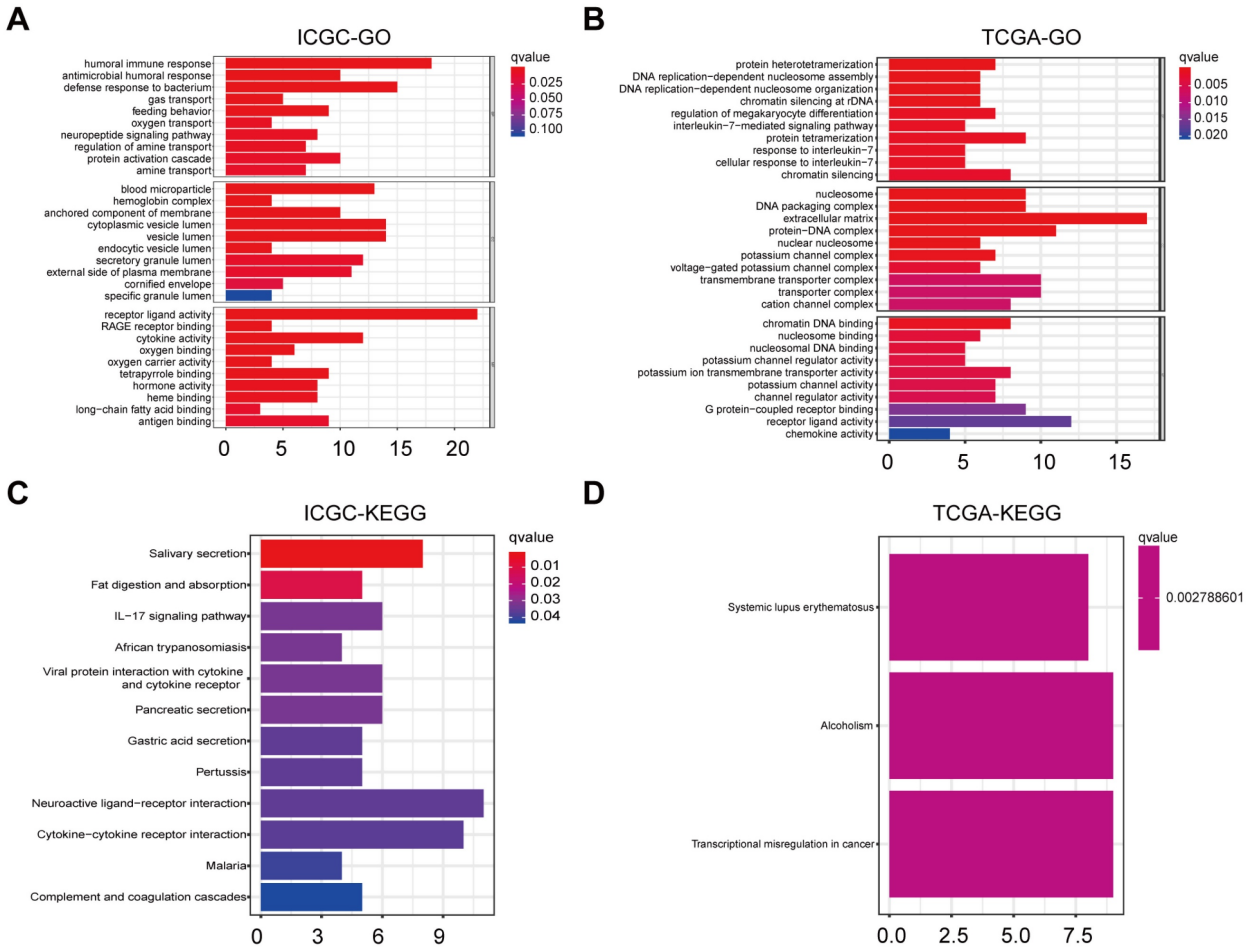
** Representative results of GO and KEGG analyses. A-D**. The most significant or shared GO enrichment and KEGG pathways in the TCGA cohort (B, D) and ICGC cohort (A, C) are displayed.

To unravel pathways and biological functions linked with the risk score, we conducted GO and KEGG analyses utilizing the DEGs identified between cohorts stratified into heightened-risk and lowered-risk categories. Within the ICGC cohort, the GO analysis revealed that the DEGs predominantly correlated with immune-related biological processes, including “humoral immune response” and “antigen binding” (*P*.adjust < 0.05, Figure 6A). These processes may indicate the immune status and response of OC patients. Within the TCGA cohort, the GO analysis unveiled a notable enrichment of DEGs in various DNA replication processes, notably including “DNA replication-dependent nucleosome assembly” and “DNA replication-dependent nucleosome organization” (*P*.adjust < 0.05, Figure 6B). These mechanisms may contribute to regulating cellular division and proliferation in OC contexts. KEGG analysis of ICGC indicates that the enrichment is related to “neuroactive ligand-receptor interaction” and so on (*P*.adjust < 0.05, Figure 6C). Likewise, KEGG analysis of TCGA suggests that the enrichment is related to “transcriptional misregulation in cancer” (*P*.adjust < 0.05, Figure 6D).

### Analysis of the expression, grade, and stage of five prognostic ferroptosis-related DEGs in OC

**Figure 7:**
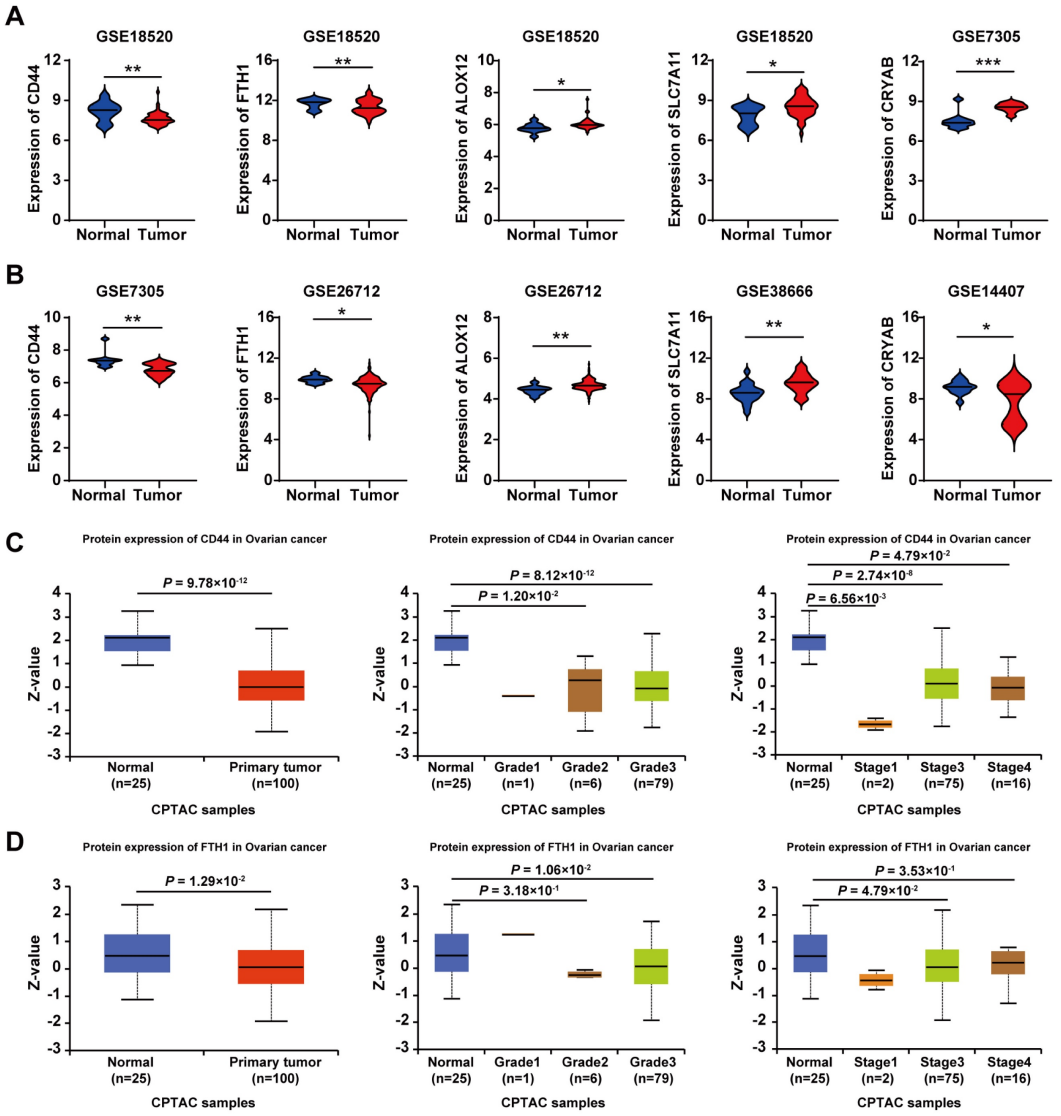
** Analysis of the expression, grade, and stage of five prognostic ferroptosis-related DEGs in OC. A-B.** Violin plots display the differential expression distribution of five key genes between normal and ovarian cancer tissues across multiple GEO datasets. **C.** Expression of CD44 in OC based on the tissue sample, tumor grade, and cancer stages. **D.** Expression of FTH1 in OC based on the tissue sample, tumor grade, and cancer stages.

Based on the five key genes (CD44, FTH1, ALOX12, SLC7A11, and CRYAB) identified through our prognostic modeling, we subsequently analyzed their expression profiles between OC tissues and normal tissues across multiple GEO datasets. As illustrated in Figure 7A-B, compared with normal controls, CD44 and FTH1 exhibited significant downregulation in OC tissues, while ALOX12, SLC7A11, and CRYAB demonstrated marked upregulation patterns. Moreover, the results from protein expression data in the UALCAN database 17 showed that, compared to normal individuals (n=25), the expression levels of CD44 and FTH1 were also significantly reduced in primary OC patients (n=100) (Figure 7C-D). Similarly, compared with normal tissues, CD44 and FTH1 expression were significantly downregulated in advanced-stage (FIGO III/IV) and high-grade (G3) ovarian cancer tissues (Figure 7C-D).

These results were consistent with our predictions using bioinformatics tools, demonstrating the reliability of the risk model constructed based on the five-gene signature.

### Correlation between gene expression and immune cell infiltration levels

**Figure 8:**
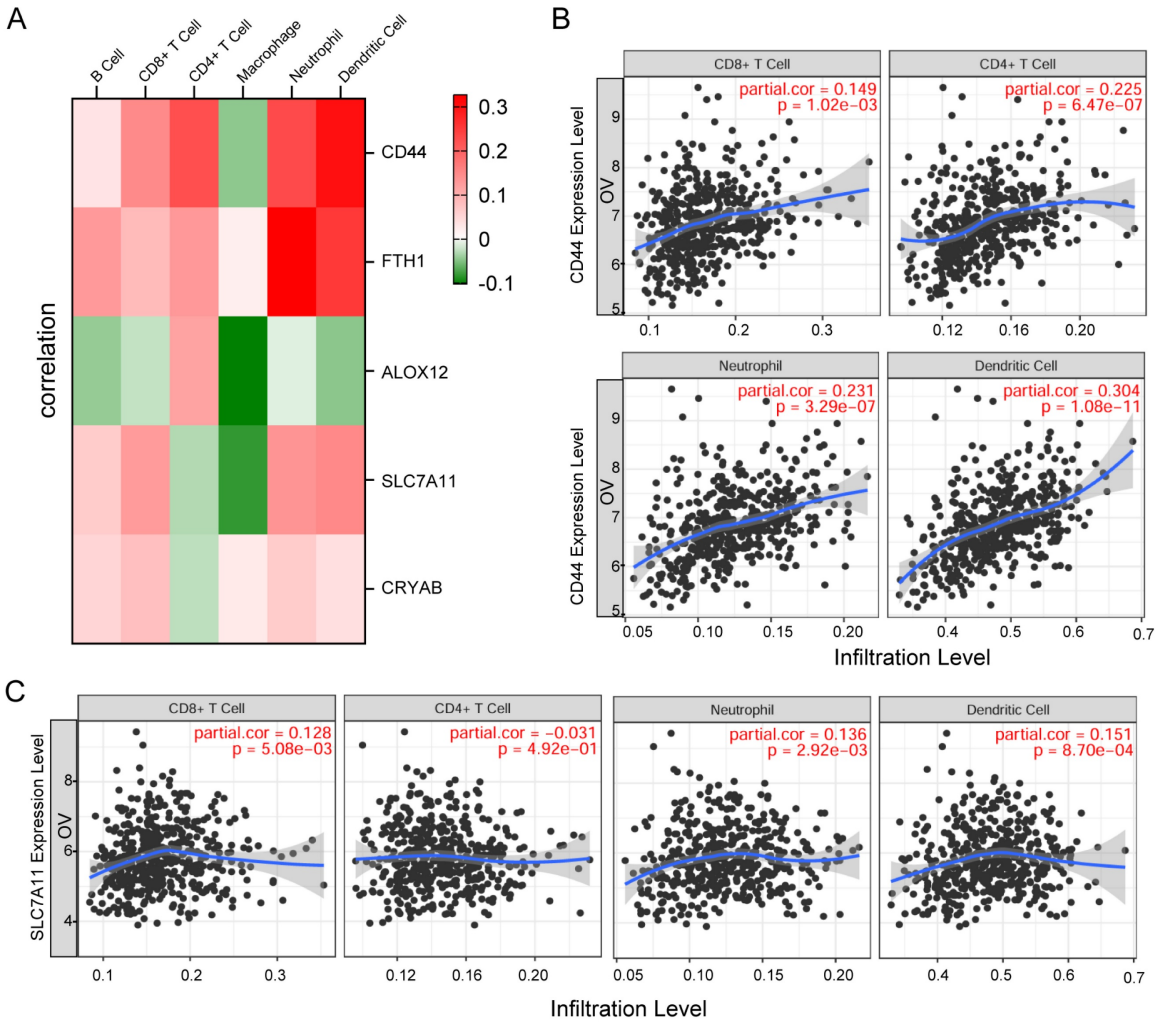
** Correlation between gene expression and immune cell infiltration levels. A.** Heatmap visualization of associations between the five genes and common immune cell types. **B-C.** The scatter plot shows the correlation between the expression of CD44, SLC7A11, and the immune infiltration in OC samples from the TIMER website.

First, the associations between these five genes and common immune cell types were visualized via heatmap analysis, and the results showed significant correlations between the five genes and key immune cell types (Figure 8A). Subsequently, we employed TIMER (Tumor Immune Estimation Resource, https://cistrome.shinyapps.io/timer/) to systematically evaluate correlations between key genes and immune cell infiltration levels in tumor tissues based on available RNA-seq data. The results demonstrated that CD44 and SLC7A11 expression in OC showed significant positive correlations with infiltration levels of CD8^+^ T cells, CD4^+^ T cells, neutrophils, and dendritic cells (all P<0.05) (Figure 8B-C).

## Discussion

**Figure 9:**
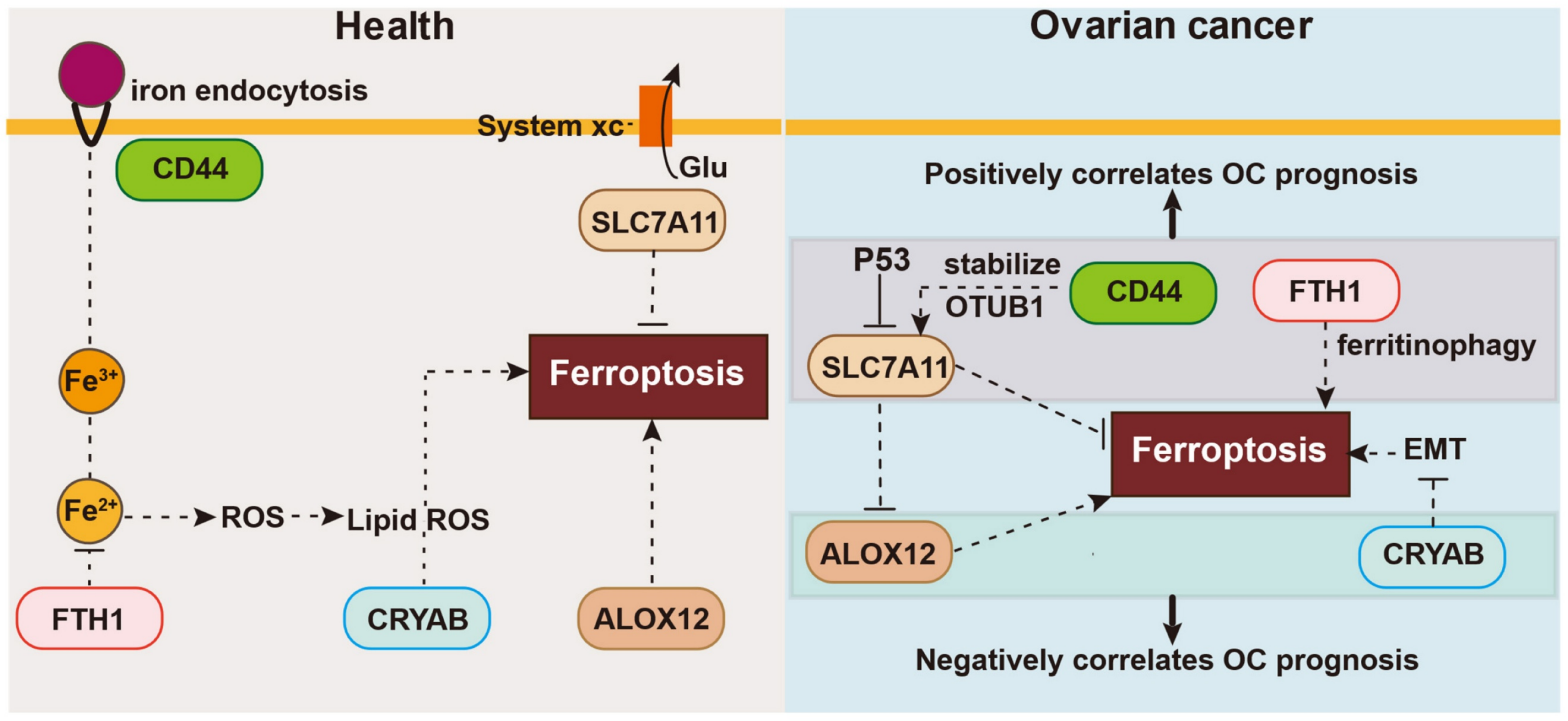
** The association between these five genes and ferroptosis in health or ovarian cancer**.

In this investigation, we performed a comprehensive analysis of the expression and predictive significance of 56 genes connected to ferroptosis in OC tumor samples and neighboring normal tissues. We developed and confirmed a forecasting model based on five genes implicated in ferroptosis in two independent cohorts. Functional analyses showed enrichment of DNA replication pathways and immune-related biological processes in the high-risk group. Preliminary research suggest that some genes may modulate ferroptosis in OC, but their association with OC prognosis is poorly understood. Here, we first explored the manifestation of ferroptosis-associated genes within OC and surrounding normal healthy tissues, finding differential expression in many genes (56 in total). Next, we selected five ferroptosis-related genes linked to OS by LASSO Cox regression analyses and univariate Cox regression. We assessed the correlation between their expression and survival, building a risk signature with the five chosen ferroptosis-related genes: CD44, FTH1, ALOX12, SLC7A11, and CRYAB. Then, consensus clustering was utilized to classify all OC samples into two clusters based on the expression patterns of all genes associated with ferroptosis. Using this signature, we constructed a nomogram integrating the prognostic data from the five genes linked with ferroptosis in OC. Univariate and multivariate statistical analyses were used to assess the prognostic significance of these five genes. Lastly, we performed OS analysis and Gene Set Enrichment Analysis (GSEA) using data from an independent OC cohort to confirm the predictive significance of the five selected ferroptosis-related genes. These results strongly highlight the potential therapeutic intervention targeting ferroptosis in OC and demonstrate the feasibility of constructing a forecasting algorithm using these five genes. The OC forecasting algorithm proposed in this research consists of five ferroptosis-linked genes: CD44, FTH1, ALOX12, SLC7A11, and CRYAB, as shown in Figure 9. The onset of ferroptosis is triggered by lipid peroxidation and tightly controlled by SLC7A11, a pivotal component of the cystine-glutamate antiporter. SLC7A11 is involved in various ferroptosis-related pathways. Moreover, research has suggested that BAP1 can induce ferroptosis by inhibiting SLC7A11 expression 18. Liu et al. reported that inactivation of the ubiquitin hydrolase OTUB1 destabilized SLC7A11, decreasing ferroptosis activation and leading to growth inhibition of tumor xenografts in mice 19. Hence, SLC7A11 is found to be upregulated in different types of human cancer, and its elevated expression can inhibit cellular ferroptosis, consequently leading to unfavorable prognostic outcomes for patients 19.

CD44 functions as an indicator of cancer stem cells and governs epigenetic plasticity by facilitating iron endocytosis 20. It also plays a role in chemotherapeutic drug resistance through ferroptosis, crosstalk, and regulatory pathways that selectively induce cancer stem cell death, exemplified by CD44, leading to improved therapeutic outcomes with certain chemotherapeutic agents. Specifically, CD44v, a variant of CD44, has the capability to stabilize the protein xCT and promote glutathione synthesis. Thus, additionally decreases ROS-triggered stress signaling, a distinguishing feature of ferroptosis 21. Moreover, Tong et al. demonstrated that heightened levels of CD44, a marker indicative of cancer stem cells, bolstered the durability of SLC7A11 by fostering the interplay between SLC7A11 and OTUB1, and mediated ferroptosis 19.

FTH1 is essential for maintaining cellular iron homeostasis during ferroptosis. FTH1 is also engaged in ferritinophagy, a specific type of autophagy 22. In the 6-OHDA model of Parkinson's disease, FTH1 induces ferroptosis via ferritinophagy 3. In agreement, FTH1 silencing in mouse intestines can lead to iron overload and facilitate ferroptosis 23, 24.

The ALOX12 gene is a frequent target of monoallelic deletion in human malignancies. Chu et al. demonstrated that ALOX12 plays a crucial role in p53-mediated tumor suppression via a distinct ferroptosis pathway, and the absence of ALOX12 can eliminate ferroptosis 25. By inactivating ALOX12, cancer cells can escape ROS-mediated ferroptosis.

In contrast to these genes, there have been fewer investigations on the signaling pathways mediated by CRYAB. CRYAB regulates multiple signaling cascades in cancer, including the PI3K/AKT and ERK pathways 26, 27. Moreover, it is linked with the initiation and progression of various malignant tumors, including gastric cancer, nasopharyngeal carcinoma, and bladder cancer, among others 28, 29. Recent studies found that CRYAB could boost FTH1 degradation and increase the Fe level, and then the ROS level, and finally improve the ferroptosis of BMSCs, with less osteogenic differentiation 30.

CD44 is linked to the mobility of tumor cells as well as the reinforcement of the interplay between OTUB1 and SLC7A11, thereby regulating the stability of SLC7A11 19. In turn, SLC7A11 inhibits ferroptosis by stimulating the synthesis of glutathione and preventing the accumulation of lipid peroxides. Furthermore, SLC7A11 directly interacts with ALOX12, suppressing the lipoxygenase activity of ALOX12 and consequently hindering ferroptosis by impeding the peroxidation pathway of unsaturated lipids. ALOX12 deficiency is commonly observed in cancer patients, and missense mutations in ALOX12 can result in the loss of its ability to oxidize polyunsaturated fatty acids, thereby enhancing p53-induced ferroptosis 31. Furthermore, ALOX12 expression restrains the growth, infiltration, and movement of cancerous cells, and additionally impedes tumor expansion in living organisms. Moreover, the expression of ALOX12 also heightens the responsiveness of tumor cells to ferroptosis initiators, consequently enhancing the frequency of tumor cell demise. The activation of signaling pathways such as p53 by ALOX12 can induce ferroptosis 32.

FTH1 is indeed regarded as a suppressor of ferroptosis. It functions by decreasing intracellular levels of free iron ions through iron ion sequestration. FTH1 also prevents lipid peroxidation, thereby effectively inhibiting the occurrence of ferroptosis 33.

In summary, our novel five-gene model not only elucidates the complex molecular mechanisms of ferroptosis in the development of OC but also serves as a useful tool for predicting patient survival. Despite the promising insights provided by our model, it is important to acknowledge the limitations associated with using public databases, emphasizing the need for future studies to explore the intricate interplay between OC and ferroptosis. By tackling these obstacles, we can lay the groundwork for more accurate and individualized treatment approaches, ultimately enhancing the quality of life and prolonging the survival of individuals grappling with OC. Our research marks a significant step forward in the ongoing efforts to unravel the complexities of OC and underscores the potential of our model in shaping the future landscape of clinical decision-making.

## Materials and Methods

### Datasets

In March 2019, we acquired RNA-seq transcriptome data from the TCGA database for 379 ovarian cancer (OC) patients along with their respective clinicopathological information (http://cancergenome.nih.gov/). Furthermore, we acquired RNA-seq transcriptome data for 88 healthy human ovarian tissues from the GTEx database 34 (https://www.gtexportal.org/home/datasets). Concerning the RNA-seq data, normalization of TCGA samples (n=379) was carried out using the fragment per kilobase of exon model per million (FPKM) method, as previously defined 35. We also applied ComBat (from the sva R package) to correct for potential batch effects. This study also utilized the UALCAN database (https://ualcan.path.uab.edu/) 17, 36, which contains protein-level data from 25 normal individuals and 100 patients diagnosed with primary ovarian cancer.

### TCGA, GTEx, UALCAN, and ICGC cohort

We retrieved RNA-seq data and clinical data of 379 OC patients from the TCGA database (http://cancergenome.nih.gov/) on September 23, 2019. We normalized gene expression profiles using the Wilcoxon test, implemented in the “limma” R package. We also obtained RNA-seq data and clinical data of 88 normal ovarian samples from the GTEx website (https://gtexportal.org/). All data from TCGA, GTEx, UALCAN, and ICGC are publicly available; thus, this study did not require local ethics committee approval. The research followed the data access policies and publication guidelines established by TCGA, GTEx, UALCAN, and ICGC. The primary focus of this study revolves around conducting a comprehensive analysis of these five genes. The accessibility of these four databases contributes to the reproducibility and verification of the research findings.

### Establishing and validating a prognostic gene signature associated with ferroptosis mechanisms

We used the Wilcoxon test to identify DEGs between OC tumor tissues and normal ovarian tissues, with a false discovery rate (FDR) < 0.05 in the TCGA cohort.

We performed univariate Cox analysis for overall survival to select ferroptosis-related genes with prognostic value. We adjusted *P* values using the Benjamini & Hochberg (BH) method. We built a network of interactive overlapping predictive DEGs using the STRING database (version 11.0) 37. To avoid overfitting in the gene signature, we developed a prognostic model 38, 39 using the DEGs with LASSO-penalized Cox regression analysis, conducted using the “glmnet” R package. We applied the LASSO algorithm to the DEGs using variable selection and shrinkage techniques provided by the “glmnet” R package. The regression model used the normalized expression matrix of candidate prognostic DEGs as the independent variable, and OS and patients' status in the TCGA cohort as the response variables. We determined the penalty parameter (λ) for the model through tenfold cross-validation, selecting the λ value corresponding to the lowest partial likelihood deviance as the minimum criterion.

We calculated patients' risk scores based on the normalized expression level of each gene and its corresponding regression coefficients. The formula is defined as follows:

score=e ^sum (univariateCoxanalysis*correspondingcoefficient)^

We stratified patients into high-risk and low-risk groups based on the median value of the risk score. We used T-distributed stochastic neighbor embedding (t-SNE) to examine the distribution of different groups using the “Rtsne” R package. Then, for each gene's survival analysis, we determined the optimal cut-off expression value using the “surv_cutpoint” function from the “survminer” R package. To evaluate the predictive power of the gene signature, we conducted time-dependent ROC curve analyses, using the “survivalROC” R package.

### Functional enrichment analysis

We performed Gene Ontology (GO) and Kyoto Encyclopedia of Genes and Genomes (KEGG) analyses on the DEGs (|log_2_FC|≥ 1, FDR <0.05) between the high-risk and low-risk groups using the “clusterProfiler” R package 40, 41. The Benjamini & Hochberg (BH) method was used to adjust *P* values. We computed the infiltration scores of 16 immune cells and 13 immune-related pathways by Single-sample Gene Set Enrichment Analysis (ssGSEA) 42 implemented in the “GSVA” R package 43. We explored the potential pathways related to ferroptosis-related genes in OC development by pathway enrichment analysis.

### Statistical analysis

We compared the gene expression between tumor tissues and normal ovarian tissues using the Student's t-test. The Chi-squared test was used to compare the proportions. The Mann-Whitney test was applied to compare the ssGSEA scores of immune cells or pathways between the high-risk and low-risk groups, with P-values adjusted by the Benjamini-Hochberg (BH) method. We compared the OS between different groups by Kaplan-Meier analysis and the log-rank test. We identified independent predictors of OS by univariate and multivariate Cox regression analyses. All the analyses were performed by R software (Version 3.5.3) or SPSS (Version 23.0). A p-value < 0.05 was considered statistically significant.
